# Deciphering the “Doxycycline Deficiency Syndrome”: Defining the Characteristic Clinical, Laboratory and Imaging Findings in Patients Presenting with Leptospirosis, Q Fever and Rickettsial Diseases in Far North Queensland, Tropical Australia

**DOI:** 10.3390/tropicalmed11090237

**Published:** 2026-08-22

**Authors:** Hayley Stratton, Callum Pownell, Megan Sandeman, Patrick Rosengren, Cody Price, Alexandra G. A. Stewart, Roderick Gavey, Toni Kinneally, Kathy Petoumenos, Simon Smith, Josh Hanson

**Affiliations:** 1 Department of Medicine, Cairns Hospital, Cairns, QLD 4870, Australia; hayley.stratton@nt.gov.au (H.S.); callum.pownell@health.qld.gov.au (C.P.); megan.sandeman@health.qld.gov.au (M.S.); toni.kinneally@health.qld.gov.au (T.K.); simon.smith2@health.qld.gov.au (S.S.); 2 School of Medicine and Dentistry, James Cook University, Cairns, QLD 4878, Australia; patrick.rosengren@my.jcu.edu.au; 3 Australian Clinical Laboratories, Clayton, VIC 3168, Australia; codyfrancisprice@gmail.com; 4 Department of Infectious Diseases, Royal Brisbane and Women’s Hospital, Brisbane, QLD 4029, Australia; alexandra.stewart03@gmail.com; 5 Faculty of Health, Medicine and Behavioural Sciences, The University of Queensland, Brisbane, QLD 4072, Australia; roderick.gavey@health.qld.gov.au; 6 The Kirby Institute, University of New South Wales, Kensington, NSW 2052, Australia; kpetoumenos@kirby.unsw.edu.au

**Keywords:** leptospirosis, Q fever, scrub typhus, Queensland tick typhus, rickettsial infections, zoonotic infections, tropical medicine, Australia

## Abstract

**Background**: The “doxycycline deficiency syndrome” is a colloquial term used in tropical Australia to describe the overlapping, non-specific findings seen in leptospirosis, Q fever, and the rickettsial diseases scrub typhus and Queensland tick typhus (QTT). A laboratory confirmed diagnosis is frequently delayed, however, early differentiation of these potentially life-threatening infections has important implications for clinical management. **Methods**: We examined consecutive laboratory-confirmed cases of leptospirosis, Q fever, and rickettsial diseases managed at a referral centre in tropical Australia. The patients’ symptoms, clinical signs and simple laboratory and radiology investigations at presentation were compared. **Results**: There were 111 cases of leptospirosis, 59 cases of Q fever, and 34 cases of rickettsial disease (17 cases of QTT and 17 of scrub typhus). Patients with leptospirosis had a shorter duration of symptoms and were more likely to have gastrointestinal symptoms, arthralgia, haemoptysis, hypotension, conjunctival suffusion and abnormal chest auscultation at presentation than patients with the other infections. They were more likely to have renal impairment and an elevated creatine kinase but less likely to have an elevated serum ferritin and serum lactate dehydrogenase. Patients with leptospirosis were more likely to have alveolar and multilobar changes on chest imaging. Patients with Q fever were more likely to have abnormal liver function tests at presentation but were more likely to have preserved renal function and normal chest imaging. Patients with rickettsial disease were more likely to have a rash, an eschar, lymphadenopathy and splenomegaly. Overall, 76/204 (37%) required ICU admission, of whom 56 (74%) had leptospirosis, but all 204 patients survived to hospital discharge. **Conclusions**: Leptospirosis, Q fever, and rickettsial diseases can have a similar presentation, but simple clinical, laboratory and imaging findings can help distinguish these infections and expedite their optimal management.

## 1. Introduction

Leptospirosis, Q fever, Queensland tick typhus (QTT) and scrub typhus are life-threatening zoonotic diseases that are endemic in tropical Australia [1,2,3]. Leptospirosis is caused by pathogenic spirochetes of the genus *Leptospira* which are excreted in the urine of infected animals, and which infect humans via skin or mucosal abrasions [4]. Q fever, caused by the gram-negative, obligate intracellular bacterium *Coxiella burnetii*, usually develops in humans after the pathogen is inhaled during contact with infected livestock, domestic mammals and wildlife, or after airborne dissemination of contaminated dust and aerosols [5]. QTT (caused by *Rickettsia australis*) and scrub typhus (caused by *Orientia tsutsugamushi*) are rickettsial infections which are transmitted to humans by the bites of hard ticks and mites respectively [6,7].

The clinical presentation of individuals with these four infections can be similar and is often non-specific [8]. Most patients will have a mild, self-limited illness, which is typically characterised by fever, malaise, myalgia and headache and laboratory abnormalities that include thrombocytopenia and abnormal liver function tests [1,6,9,10]. But delays in the initiation of appropriate therapy for these pathogens can result in a more severe illness, necessitating a requirement for hospitalisation and, in some, intensive care unit (ICU) admission. Even in Australia’s well-resourced health system, deaths from all four infections have been reported [11,12,13].

The similarities in the epidemiology and the clinical presentation of these zoonotic infections and the response of all four pathogens to doxycycline explain why Australian clinicians colloquially describe these patients, collectively, as having a “doxycycline deficiency syndrome”. However, distinguishing the infections is also important because their acute clinical course and management can differ significantly.

Individuals with all four infections can have respiratory symptoms, but even subtle evidence of lung involvement may be a harbinger of fatal pulmonary haemorrhage in leptospirosis [14]. Individuals with leptospirosis can also develop a Jarisch–Herxheimer reaction after initiation of antibiotics necessitating more immediate delivery of supportive care [15]. Adjunctive therapies, including corticosteroids, may have a role in the early treatment of leptospirosis, although their utility is incompletely defined [16,17,18]. Patients with acute Q fever can develop potentially lethal cardiac, haematological and neurological complications which may not be immediately apparent and which can be challenging to treat [19,20]; they also need close follow up to exclude the development of chronic Q fever which has a case-fatality rate that exceeds 20% in contemporary series [21,22]. Meanwhile, some authors have argued that patients with severe scrub typhus should receive combination therapy with doxycycline and azithromycin rather than doxycycline monotherapy [23].

But how can clinicians distinguish these pathogens? Classic manifestations of the individual infections are described and include conjunctival suffusion in leptospirosis and an eschar and rash in rickettsial diseases, but these characteristic findings are not present in all cases, and even experienced health workers working in endemic settings have difficulty differentiating the pathogens clinically [8,24]. It is also almost never possible to have a laboratory confirmed diagnosis at a time when this can inform immediate clinical care. Reliable point of care tests are not available, serology for all four infections is frequently negative at the time of presentation, and the results of molecular testing are rarely available promptly in the rural locations where these infections are encountered most commonly [3,25,26,27].

This retrospective study examined individuals with a laboratory confirmed diagnosis of leptospirosis, Q fever, QTT and scrub typhus at a referral hospital in tropical Australia. The study compared the symptoms and signs, the simple laboratory and radiological findings of each of the infections at presentation and their subsequent course to define their clinical phenotypes more precisely. There was a particular focus on determining the findings that were more—or less—likely to be associated with each of these pathogens. The study aimed to generate data that could be used in locations where these infections are endemic to expedite their recognition and facilitate their optimal management while a definitive diagnosis is awaited.

## 2. Methods

This retrospective study was performed at Cairns Hospital, the referral hospital for the region of Far North Queensland (FNQ). The hospital has 771 beds and serves a population of ~290,000 people who live across a predominantly rural, geographical area of 380,000 km^2^ in remote tropical Australia (Figure S1). The FNQ region includes the city of Cairns (an international tourist destination and FNQ’s administrative hub) but it also includes the Cassowary Coast region (home to farms that produce 80% of Australia’s bananas and large quantities of sugar cane) and the Atherton Tablelands which also has a busy agricultural sector and a thriving dairy cattle industry [28].

Individuals were eligible for inclusion in the study if they were admitted to Cairns Hospital and had a laboratory confirmed diagnosis of leptospirosis, acute Q fever, QTT or scrub typhus. The Australian definitions of laboratory confirmed leptospirosis and acute Q fever are presented in Table S1. Confirmed QTT or scrub typhus infection was defined as either a positive polymerase chain reaction (PCR) test or four-fold rise in titres of paired serological specimens. Details of the PCR methodology that was used to diagnose the four pathogens are presented in Files S1–S4. The study included cases that have been presented in cohorts describing the clinical course of these infections previously [2,10,25].

Eligible individuals were identified using the Queensland public health system’s electronic laboratory database (AUSLAB) and included every case of laboratory-confirmed leptospirosis or acute Q fever, between 1 January 2015 and 30 June 2024; this study period was chosen to coincide with the introduction of an electronic medical record system at the hospital. Individuals with a laboratory-confirmed diagnosis of QTT or scrub typhus admitted to Cairns Hospital between 1 June 1997 to 30 June 2024 were also eligible for inclusion. The combination of these rickettsial infections and the longer eligibility period was chosen to increase the number of cases of rickettsial disease in the cohort, allowing meaningful comparison with the other infections.

The patients’ demographics, relevant exposures (occupational and recreational) and comorbidity information (defined in Table S2) were collected from their medical records. Findings at presentation (defined as the symptoms, clinical signs and laboratory results that were recorded on admission to Cairns Hospital) were also recorded. A patient was said to have a biphasic presentation if there was a >24 h period of defervescence or diminution in symptoms prior to presentation [29]. Chest imaging findings were recorded using the specialist radiologist reports that were available on the hospital’s electronic imaging system. The medical record was reviewed to determine if the admitting clinician named one of the pathogens or included “zoonotic infection” in their initial differential diagnosis. The length of hospitalisation, the requirement for ICU admission and all-cause mortality before hospital discharge was also recorded.

### Statistical Analysis

De-identified data were entered into an electronic database (Microsoft Excel, version 16.0) and analysed with statistical software (Stata version 18.5). Groups were analysed using the Kruskal-Wallis test, the chi-squared test and Fisher’s exact test, where appropriate. Logistic regression was used to quantify the association between the variables most associated with the different infections. If individuals were missing data, they were not included in analyses which evaluated those variables.

## 3. Results

There were 204 individuals who satisfied eligibility criteria for the study, this included 111 cases of leptospirosis, 59 cases of Q fever, and 34 cases of rickettsial disease (17 cases of QTT cases and 17 cases of scrub typhus, respectively). The patients’ median (interquartile range, IQR) age was 45 (29–60) years, 159/204 (78%) were male and 160/204 (78%) had no recorded comorbidity (Figure S2, Table S3).

Individuals with leptospirosis were younger (odds ratio (OR) 95% confidence interval (CI): 0.97 (0.96–0.99), *p* = 0.001) and less likely to have comorbidity (OR (95% CI: 0.27 (0.13–0.55), *p* < 0.001) than the individuals with the other infections.

### 3.1. Geographical, Occupational and Recreational Risk Factors

Of the 204 cases, 131 (64%) were transferred to Cairns Hospital from another health facility. Residents of the Tablelands region, home to the region’s intensive cattle farming, were more likely to have Q fever than the other infections: 22/53 (42%) Tablelands residents had Q fever versus 37/151 (25%) individuals living in other regions, *p* = 0.02. A greater proportion of residents of the Cassowary Coast (home to the region’s intensive banana and sugar farming industry) had leptospirosis, but the difference did not reach statistical significance: (36/56 (64%) Cassowary Coast residents had leptospirosis versus 75/148 (51%) individuals living in other regions, *p* = 0.08) (Figure S3).

In the cohort of 204, 44 (22%) worked as a farmer (37/111 (33%) cases of leptospirosis, 6/59 (10%) cases of Q fever and 1/34 (3%) cases of rickettsial disease). A history of freshwater exposure was recorded in 23/204 (11%): 22/111 (20%) cases of leptospirosis, 1/59 (2%) cases of Q fever cases, but in no cases of rickettsial disease.

### 3.2. Diagnosis

PCR confirmed the diagnosis of leptospirosis in 89/111 (80%) and Q fever in 45/59 (76%). Rickettsial diseases were diagnosed more commonly with paired serology (26/34, 76%) (Table S4). In most cases the results of microbiological testing were not available until >72 h after these tests were requested (Table 1).

One of the four pathogens was named, or “zoonotic infection” was included in the differential diagnosis of the admitting medical officer in 82/204 (40%) presentations: 55/111 (50%) cases of leptospirosis, 5/59 (8%) cases of Q fever and 22/65 (50%) of the rickettsial infections (Table 2).

### 3.3. Clinical Presentation

#### 3.3.1. Symptoms

Individuals with leptospirosis had a shorter duration of symptoms prior to presentation than individuals with the other infections (Table 3, Table 4 and Table 5). None of the cases of leptospirosis had symptoms in a biphasic pattern.

Fever was the most common presenting subjective symptom for all four infections. Fatigue and rigors were more common in patients with Q fever, while gastrointestinal symptoms and arthralgia were more common in leptospirosis. Haemoptysis was only reported in patients with leptospirosis (Table 3).

#### 3.3.2. Signs

Conjunctival suffusion was only documented in leptospirosis cases, and an eschar was identified in only one individual who did not have a rickettsial disease, but both signs were present in the minority of individuals with these respective diagnoses. Hypotension and abnormal findings on chest auscultation were more common in cases of leptospirosis. A rash, lymphadenopathy and splenomegaly were more commonly documented in cases of rickettsial disease. This lymphadenopathy was frequently in association with an eschar (odds ratio (95% confidence interval): 9.0 (2.6–31.6). There were no examination findings that were associated with an increased likelihood of Q fever (Table 4, Table 5 and Table 6).

### 3.4. Simple Laboratory Investigations

The white blood cell and neutrophil count were higher in patients with leptospirosis at presentation than in patients with the other infections; the platelet count was similar for all infections. Individuals with leptospirosis were more likely to have renal impairment and an elevated creatine kinase (CK) at presentation. In contrast, they were less likely to have liver function test derangement, an elevated lactate dehydrogenase (LDH) or an elevated ferritin at presentation than individuals with Q fever and rickettsial disease (Table 7). These trends persisted during the patients’ subsequent hospitalisation (Table S5). Every patient with Q fever had an abnormal alanine aminotransferase (ALT) at presentation.

### 3.5. Chest Imaging

A greater proportion of individuals with rickettsial disease had abnormal chest imaging at presentation than those with the other infections. Individuals with rickettsial disease were more likely to have interstitial changes on chest imaging. Individuals with leptospirosis were more likely to have alveolar changes and multilobar involvement (Table 4, Table 5 and Table 8).

### 3.6. Clinical Course and Outcomes

Antibiotic therapy with activity against the pathogen was prescribed in 201/204 (99%) individuals. It was possible to determine the timing of antibiotic commencement in 196/201 (98%). Effective antibiotic therapy was commenced on the first day of hospitalisation in 167/196 (85%) and was more likely to be commenced on the first day of hospitalisation in patients in whom a zoonotic infection was in the initial differential diagnosis than in those in whom it was not (77/79 (97%) versus 90/117 (77%), *p* < 0.001).

Overall, 76/204 (37%) required ICU admission; in only 34/76 (45%) was a zoonotic infection in the initial differential. Patients with leptospirosis were more likely to require ICU admission, vasopressor support and renal replacement therapy (RRT) than individuals with the other infections. Individuals with Q fever were less likely to require any organ support. Individuals with rickettsial diseases had a longer hospital stay. All patients in the cohort survived to hospital discharge (Table 9).

## 4. Discussion

Leptospirosis, Q fever and rickettsial infections usually have an uncomplicated course if they are recognised promptly and managed appropriately. However, it was notable that although all four pathogens are endemic in the FNQ region, in almost 60% of the cases of this cohort the admitting clinician’s initial differential diagnosis did not include a zoonotic infection. Appropriate antibiotic therapy was initiated later in patients in whom this diagnosis was not considered, which has been linked to poorer outcomes in all four infections [30,31,32,33]. While there were no deaths in the cohort, over 37% required ICU admission to survive their infection.

Pragmatists may argue that the recognition of the clinical syndrome is the main challenge as the acute management of the four infections is similar and includes prompt antibiotic therapy, supportive care, and where necessary, ICU admission for organ support. However, we would argue it is important to not only recognise the infections, but also to distinguish them, so that clinicians can anticipate potentially fatal complications of the individual infections, expedite specific investigations to inform their optimal clinical management and to reduce the health system costs of unnecessary diagnostic evaluation [34,35,36].

FNQ has the highest incidence of leptospirosis in Australia which may explain why it was the infection that was most likely to be recognised at presentation [1,37]. Patients with leptospirosis were younger and had less comorbidity, reflecting the demography of workers in the local banana and sugar cane industries and individuals enjoying recreational activities in freshwater. It was notable that the duration of symptoms among individuals with leptospirosis prior to presentation was also relatively short and was not biphasic, at odds with the classic teaching, and suggesting that this finding is not helpful in identifying cases of leptospirosis [38].

Clinical findings in the patients with leptospirosis had only modest predictive value. While conjunctival suffusion was highly specific, it was only reported in 21% of the patients. Myalgia, another classically described finding in leptospirosis, was as common in patients with rickettsial infection, although serum CK levels were much higher in the patients with leptospirosis and could be explained by muscle damage from direct organism invasion [39,40]. Although “severe disease” and “icteric disease” are often used interchangeably in patients with leptospirosis [4,41], it was notable among the patients with leptospirosis admitted to this referral hospital, more than half of whom required ICU admission, that the serum bilirubin at presentation and during the hospitalisation was no greater than that seen in patients with the other infections [42]. This observation echoes previous series from Australia and may be explained by the fact that infection with serovars from Icterohaemorrhagiae serogroup are less commonly seen in Australia than in other parts of the world [1,25,30,43,44,45,46,47].

Individuals with leptospirosis were more likely to have a history of haemoptysis and an abnormal respiratory examination at presentation, which is likely to be explained by the unique pathophysiology of lung involvement in patients with leptospirosis. The organism causes damage to lung capillaries, leading to loss of vascular integrity and alveolar oedema and haemorrhage, which is exacerbated by a reduction in the sodium transport capacity of alveolar epithelial cells, further impairing pulmonary fluid handling [48,49,50,51]. These pathophysiological changes are also likely to explain the finding that alveolar changes were more common on chest imaging of the individuals with leptospirosis during the patients’ hospitalisation.

Individuals with leptospirosis were also more likely to have impaired renal function at presentation and during their hospitalisation. No fewer than 16% of the patients with leptospirosis required RRT during their hospitalisation. Leptospirosis causes renal impairment through a variety of mechanisms that include tubulointerstitial nephritis, and acute tubular necrosis [52,53]. *Leptospira* can be identified adhering to the epithelial surface of the renal tubules and in the tubular lumen causing local inflammation and tubular dysfunction [53]. However, there is also commonly a significant contribution to the observed renal impairment by pre-renal factors including hypotension. The precise mechanism of hypotension in leptospirosis is incompletely understood, but invasive haemodynamic monitoring demonstrates low systemic vascular resistance, as is seen in sepsis [54,55]. Hypovolaemia from gastrointestinal losses and poor oral intake contribute in many (over 80% of the patients with leptospirosis in this cohort had gastrointestinal symptoms), while in some cases myocarditis plays a significant role [56].

The incidence of Q fever in the FNQ region is one of the highest in Australia, so it was notable that a zoonotic infection was considered in less than 10% of those subsequently diagnosed with *C. burnetii* infection [57]. This is important given the specific antibiotic therapy which is recommended in patients with acute Q fever, and the morbidity associated with delayed therapy or a missed diagnosis [22,32]. Almost 75% of cases of Q fever in this cohort occurred in males who were older than the patients with the other infections, reflecting the demographics of the cattle farmers in the region [58]. Fatigue was a common symptom, present in over 80%, although this may be explained by a more subacute presentation than was seen in the other infections. Patients with Q fever had the greatest derangement of their liver function tests during their hospitalisation, which is likely to be explained by the granulomatous hepatitis which is a characteristic and common finding in patients with acute Q fever [20,59,60]. However, they were far less likely to have significant renal impairment, an observation that has been noted in other cohorts and which may be due to a lack of renal tropism and less frequent contribution from pre-renal factors [20,61,62]. Chest imaging was also more likely to be normal in individuals with Q fever at presentation and during their hospitalisation, a finding that echoes previous Australian cohorts where respiratory involvement is a less frequent finding than is seen in other countries [9,59].

Patients with rickettsial infections classically present with the triad of fever, headache, and a petechial or maculopapular rash [63]. In this cohort, it was the presence of a rash—documented in over 80% of the patients with a rickettsial infection—which was the most helpful clue to this diagnosis and explained by the vasculitis of small and medium blood vessels that is the pathological signature of rickettsial infections [63,64]. The presence of an eschar had greater specificity than the presence of a rash, and therefore greater positive predictive value, but its utility was limited by the fact that it was identified in less than 40% of patients with a rickettsial infection in the cohort. There is significant variation in the presence of an eschar in international series of patients with rickettsial infection, although this is likely to reflect, at least in part, the diligence with which this important clinical sign is sought [65,66]. The presence of lymphadenopathy—which was associated with the presence of an eschar in our cohort—should prompt the clinician to examine the region thoroughly for an otherwise unidentified eschar [67,68].

Our data suggest that an elevated LDH and ferritin should also, in the appropriate clinical context, encourage clinicians to consider the diagnosis of a rickettsial infection or Q fever. The strikingly elevated LDH and ferritin in the patients with rickettsial infection might be explained by the massive cytokine release seen in severe rickettsial disease [63,69,70,71], while in acute Q fever it might be explained by the importance of the macrophage in the lifecycle of *C. burnetii* [72,73]. The presence of an elevated LDH and ferritin may also prompt the consideration of hemophagocytic lymphohistiocytosis (HLH), a rare complication of *C. burnetii* and rickettsial infections, although no patients in our cohort received HLH-specific therapy and all survived to discharge [20,74,75]. It was notable that while most patients were thrombocytopenic at presentation, the degree of thrombocytopenia was unrelated to the underlying pathogen and coagulopathy was uncommon.

Our study has many limitations. Clinical data were not collected prospectively or in a standardised manner; specific symptoms and signs was determined by retrospective review of the medical record and relied upon thorough clinical assessment and, as importantly, documentation. Prior to January 2015, paper medical records were used, which sometimes precluded complete data collection in the patients with rickettsial infection. The study was performed at a referral hospital and therefore included a sicker cohort of patients; our findings are likely to be less generalisable to individuals with milder disease. Including only laboratory confirmed cases enhances the reliability of our findings but again will tend to include patients with more severe disease in whom the diagnosis is sought more assiduously. It certainly underestimates the true local incidence of all four pathogens. There were fewer individuals with rickettsial disease and Q fever in the cohort, explained at least in part by less familiarity with the conditions and the completeness of the patients’ diagnostic workup, but this also limited the statistical power of the comparisons. The cohort included individuals with rickettsial disease from before 2000, who presented more than 15 years earlier than the patients with leptospirosis and Q fever. Improvements in medical care, enhanced retrieval and advances in critical care may have translated into a less complicated course for the patients with leptospirosis and Q fever enrolled more recently [76,77]. Residential address was used to define the different regions, which does not necessarily reflect where the individual contracted the infection. Microbiologists may take issue with our conflating the results of the patients with QTT and scrub typhus, however, the infections have a similar pathophysiology and so we felt it was reasonable that from a pragmatic perspective to consider them together [10]. The study only compared leptospirosis, Q fever and rickettsial diseases, there are clearly many other infections (including malaria, dengue and typhoid) and non-communicable conditions (including vasculitis and thrombotic microangiopathy syndromes) that can have a similar non-specific presentation, and which should be considered in the appropriate clinical context [41]. While our findings may help clinicians in tropical Australia, differences in the incidence of these pathogens mean they may have less utility in other geographic locations. In other locations the “doxycycline deficiency state” may include other conditions including Lyme disease, human ehrlichiosis and anaplasmosis [78]. Geographic heterogeneity in the presentation of leptospirosis, Q fever and rickettsial diseases is also well described [20,45,79,80,81].

Acknowledging these limitations, the study provides insights into the characteristic presentations of individuals with these zoonotic diseases in this region of tropical Australia. These clinical presentations can, in many cases, be explained by the pathophysiology of the infections and will assist local clinicians in rational and cost-effective patient management. Specifically, they may expedite the recognition of potentially fatal complications of the acute illness [14,19,20], and in some cases, inform antibiotic therapy [23]. They can ensure that individuals receive optimal screening at baseline for long term complications and ensure enrolment in longitudinal follow up [21,82]. Early identification of an outbreak of any of the pathogens may also expedite recognition of subsequent cases and ensure that they receive prompt therapy [83,84,85]. Future prospective studies could compare clinical and laboratory data that are collected in a more systematic manner. The ongoing evolution in diagnostic testing—particularly point of care testing—would also be expected to facilitate patient recognition and public health surveillance of these globally distributed infections [27].

## 5. Conclusions

Leptospirosis, Q fever, and rickettsial diseases can have a similar presentation, but simple clinical, laboratory and imaging findings can help clinicians identify and distinguish these infections and ensure their optimal management. These findings may be particularly helpful for clinicians working in rural and remote regions and resource-limited settings where these life-threatening infections are encountered most commonly and where a laboratory confirmed diagnosis is rarely available to inform acute clinical care.

## Figures and Tables

**Table 1:** Turnaround time for the diagnostic tests that were used in the cohort ^a^.

Diagnostic Test	Median (IQR) Duration (Days) ^b^
Leptospirosis PCR	4.2 (3.1–5.9)
Leptospirosis serology	3.7 (2.8–4.9)
Q fever PCR	3.1 (2.1–4.9)
Q fever serology	3.2 (2.3–4.8)
Rickettsia species PCR	2.1 (1.8–2.4)
Rickettsia serology	4.0 (2.9–5.2)
*Orientia tsutsugamushi* PCR	4.4 (3.2–6.3)
Scrub typhus serology	4.0 (2.6–5.1)

IQR: interquartile range; PCR: polymerase chain reaction. ^a^ Time from the specimen’s collection to the electronic publication of a validated result. ^b^ Data were not available for all tests for the entire study period.

**Table 2:** Initial diagnosis of the admitting clinician at the time of the patient’s presentation to hospital.

	Leptospirosis	Q Fever	Rickettsial Diseases ^a^	All
Zoonotic infection ^b^	55 (50)	5 (8)	22 (65)	82 (40)
“Febrile illness”	1 (1)	27 (46)	-	28 (14)
“Sepsis” ^c^	14 (13)	4 (7)	2 (6)	20 (10)
“Viral illness”	7 (6)	7 (12)	4 (12)	18 (9)
“Tropical infection” ^d^	10 (9)	2 (3)	-	12 (6)
Pneumonia	5 (5)	3 (5)	1 (3)	9 (4)
Arboviral infection	4 (4)	1 (2)	2 (6)	7 (3)
Autoimmune disease	3 (3)	1 (2)	2 (6)	6 (3)
Cholangitis/cholecystitis	1 (1)	5 (8)	-	6 (3)
Meningitis	4 (4)	-	-	4 (2)
Urinary tract infection	3 (3)	-	-	3 (1)
“Atypical infection” ^d^	1 (1)	-	1 (3)	2 (1)
Gastroenteritis	1 (1)	1 (2)	-	2 (1)
Acute coronary syndrome	1 (1)	-	-	1 (0.5)
Acute kidney injury	1 (1)	-	-	1 (0.5)
Acute hepatic failure	-	1 (2)	-	1 (0.5)
Shock (unspecified)	-	1 (2)	-	1 (0.5)
No diagnosis proffered	-	1 (2)	-	1 (0.5)
Total	111 (100)	59 (100)	34 (100)	204 (100)

Absolute number (%) presented. ^a^ Includes individuals with confirmed Queensland tick typhus and scrub typhus. ^b^ “Zoonotic infection” or leptospirosis, Q fever or rickettsial disease specifically documented in the admitting clinician’s differential diagnosis. ^c^ No primary site suggested. ^d^ While this might include one of the zoonotic infections, other locally prevalent tropical or “atypical” infectious diseases could be implied.

**Table 3:** Subjective symptoms reported by patients at presentation.

Variable	Leptospirosis *n* = 111	Q Fever *n* = 59	Rickettsial Diseases ^a^ *n* = 34	*p*
Symptom duration (days)	4 (3–5)	7 (5–10)	7 (6–10) ^b^	0.0001
Headache	80 (72)	35 (59)	19 (56)	0.10
Fevers	106 (96)	58 (98)	34 (100)	0.62
Rigors	40 (36)	32 (54)	9 (26)	0.02
Confusion	8 (7)	7 (12)	3 (9)	0.56
Fatigue	43 (39)	48 (81)	18 (53)	<0.0001
Abdominal pain	42 (38)	13 (22)	1 (3)	<0.0001
Myalgia	83 (75)	30 (51)	24 (71)	0.006
Arthralgia	48 (43)	16 (27)	1 (3)	<0.0001
Diarrhoea	41 (37)	10 (17)	0	<0.0001
Nausea/vomiting	74 (67)	23 (39)	18 (58)	0.002
Any GI symptoms ^c^	90 (81)	35 (59)	19 (56)	0.001
Chest pain	9 (8)	3 (5)	0	0.22
Dyspnoea	16 (14)	10 (17)	5 (15)	0.93
Cough	33 (30)	16 (27)	14 (41)	0.34
URTI symptoms	15 (14)	7 (12)	0	0.049
Haemoptysis	12 (11)	0	0	0.004
Bleeding/bruising	11 (10)	3 (6)	0	0.11

Absolute number (%) or median (interquartile range) presented. GI: gastrointestinal; URTI: upper respiratory tract infection. ^a^ Includes individuals with confirmed Queensland tick typhus and scrub typhus. ^b^ One individual with scrub typhus did not have a duration of symptoms documented. ^c^ Includes abdominal pain, nausea, vomiting or diarrhoea.

**Table 4:** Summary of the important differences found between individuals with leptospirosis, Q fever and rickettsial diseases in the cohort.

	Leptospirosis	Q Fever	Rickettsial Disease ^a^
Presenting symptoms	Shorter duration of symptoms before presentation More gastrointestinal symptoms and arthralgia; haemoptysis	More rigors and fatigue	
Initial examination findings	More likely to have hypotension, conjunctival suffusion or abnormal chest auscultation Less likely to be febrile.		More likely to have skin rash, eschar, lymphadenopathy or splenomegaly
Initial laboratory investigations	Higher white cell count, neutrophil count. Higher CK and CRP Less liver function derangement Lower LDH and ferritin	All had abnormal ALT Preserved renal function High LDH and ferritin	High LDH and ferritin
Subsequent laboratory investigations	Higher peak WCC and neutrophil count, CK and CRP Greater renal impairment	More liver function test derangement (highest peak ALT and AST)	Higher peak APTT Highest peak lactate
Chest imaging	Multilobar and alveolar changes more common	Chest imaging more commonly normal	More abnormal imaging on admission and during hospitalisation Interstitial abnormalities more common
Clinical course	More likely to require ICU admission, vasopressors and RRT		Longer hospital admission, longer ICU stay More likely to require intubation and mechanical ventilation

ICU: Intensive care unit; ALT: alanine transaminase; AST: aspartate aminotransferase; WCC: white cell count; RRT: renal replacement therapy; LDH: lactate dehydrogenase; CK: creatinine kinase; CRP: c-reactive protein; APTT: activated partial thromboplastin time. ^a^ Includes individuals with confirmed Queensland tick typhus and scrub typhus.

**Table 5:** Odds ratios for selected clinical and laboratory findings, stratified by pathogen.

Pathogen	Variable ^a^	Odds Ratio	95% Confidence Interval
Leptospirosis	Conjunctival suffusion ^b^	ꝏ	-
	Haemoptysis ^b^	ꝏ	-
	<7 days of symptoms	12.2	6.1–24.4
	Normal LDH ^c^	10.4	3.1–35.5
	Hypotension ^d^	5.4	2.7–10.7
	Elevated creatine kinase ^e^	4.3	1.9–9.7
	Abnormal chest auscultation	3.4	1.7–6.7
	Elevated white cell count ^f^	3.4	1.6–7.0
	Gastrointestinal symptoms ^g^	3.1	1.7–5.8
Q fever	History of fatigue	6.0	2.9–12.5
	Normal creatinine ^h^	4.3	2.1–8.9
	Normal initial chest imaging	3.3	1.5–7.3
	Elevated ALT ^i^	ꝏ	-
Rickettsial disease ^j^	Eschar identified	104.6	13.0–840.7
	Rash present	35.0	12.9–94.9
	Splenomegaly	22.5	2.4–208.6
	Lymphadenopathy	11.4	3.8–34.2

ꝏ: Impossible to determine odds ratio as only patients with this pathogen had this finding, or in the case of an abnormal ALT at presentation, although many patients with leptospirosis and rickettsial disease had an abnormal ALT at presentation, **every** patient with Q fever had an abnormal ALT at presentation. LDH: lactate dehydrogenase; ALT: alanine transferase. ^a^ At initial assessment. ^b^ Every patient with conjunctival suffusion or haemoptysis had leptospirosis. ^c^ Less than 250 IU/L. ^d^ Systolic blood pressure < 100 mmHg (only individuals ≥ 16 years of age) or a requirement for vasopressors. ^e^ Greater than 145 IU/mL. ^f^ Greater than 11 × 10^9^/L. ^g^ Abdominal pain, nausea, vomiting or diarrhoea. ^h^ Less than 110 µmol/L. ^i^ Every patient with Q fever had an abnormal ALT at presentation. ^j^ Includes individuals with confirmed Queensland tick typhus and scrub typhus.

**Table 6:** Objective examination findings on presentation.

Variable	Leptospirosis *n* = 111	Q Fever *n* = 59	Rickettsial Disease ^a^ *n* = 34	*p*
Hepatomegaly	11 (10)	14 (24)	5 (15)	0.06
Splenomegaly	0	1 (2)	4 (12)	0.001
Lymphadenopathy	6 (5)	0	10 (29)	<0.0001
Eschar	0	1 (2)	13 (38)	<0.0001
Conjunctival suffusion	23 (21)	0	0	<0.0001
Skin rash	19 (17)	1 (2)	28 (82)	<0.0001
Abnormal chest auscultation	44 (40)	15 (25)	0	<0.0001
Fever ≥ 38.0° Celsius	21 (19)	35 (59)	19 (56)	<0.0001
Supplemental oxygen administered	23 (21)	7 (12)	4 (12) ^b^	0.29
Respiratory rate ≥ 22 beaths/minute	44 (40)	23 (40) ^c^	21 (62)	0.06
Heart rate ≥ 100 beats/minute	54 (49)	27 (47) ^c^	22 (65)	0.20
Systolic blood pressure < 100 mmHg ^d^	56 (50)	7 (12) ^c^	10 (29)	<0.0001

Absolute number (%) presented. ^a^ Includes individuals with confirmed Queensland tick typhus and scrub typhus. ^b^ In one individual with scrub typhus it was not possible to determine if supplemental oxygen was delivered. ^c^ In one individual with Q fever, it was not possible to determine the respiratory rate, the heart rate or the blood pressure at admission. ^d^ Or vasopressor use.

**Table 7:** Haematological and biochemical values on presentation to hospital.

		Leptospirosis		Q Fever		Rickettsial Disease ^a^		*p*
Variable	Reference Range	Number with Data	All *n* = 111	Number with Data	All *n* = 59	Number with Data	All *n* = 34	
Haemoglobin	115–160 g/dL	111	134 (111–147)	59	142 (129–154)	34	129 (123–148)	0.02
White cell count	4.0–11.0 × 10^9^/L	111	9.3 (6.8–11.9)	59	5.8 (4.2–6.7)	34	7.6 (6.5–11.6)	0.0001
Platelet count	140–400 × 10^9^/L	111	119 (72–165)	59	103 (75–149)	34	107 (63–155)	0.90
Neutrophil count	2.0–8.0 × 10^9^/L	111	8.2 (5.5–10.6)	58	4.2 (2.8–5.2)	34	7.0 (4.9–10.6)	0.0001
Lymphocyte count	1.0–4.0 × 10^9^/L	111	0.5 (0.3–0.7)	58	1.0 (0.6–1.3)	34	0.6 (0.4–1.0)	0.0001
INR	0.9–1.2	85	1.1 (1.1–1.3)	35	1.2 (1.1–1.3)	23	1.2 (1.1–1.2)	0.27
APTT	25–38 s	85	31 (29–34)	33	32 (29–36)	25	33 (31–39)	0.03
Serum sodium	135–145 mmol/L	111	133 (129–135)	59	132 (128–135)	33	132 (128–135)	0.59
Serum potassium	3.5–5.2 mmol/L	111	3.7 (3.4–4.0)	59	3.9 (3.6–4.1)	30	4.0 (3.6–4.3)	0.0006
Serum urea	2.5–7.8 mmol/L	111	6.4 (4.8–12.3)	59	5.8 (4.5–7.8)	34	6.6 (4.3–11.8)	0.11
Serum creatinine	45–90 µmol/L	111	113 (88–205)	59	87 (76–104)	34	100 (80–145)	0.0001
Serum bicarbonate	22–32 mmol/L	111	23 (21–25)	59	25 (23–26)	33	23 (20–25)	0.0006
Serum bilirubin	<20 µmol/L	111	18 (12–28)	59	18 (14–28)	34	21 (13–28)	0.82
Serum ALT	<34 IU/mL	111	68 (27–115)	59	119 (80–222)	34	108 (88–140)	0.0001
Serum AST	<31 IU/mL	111	63 (34–135)	59	129 (81–259)	34	156 (99–214)	0.0001
Serum GGT	<38 IU/mL	111	51 (22–120)	59	92 (43–179)	34	86 (45–192)	0.007
Serum SAP	30–110 IU/mL	111	98 (67–171)	59	98 (78–203)	34	131 (77–220)	0.10
Serum LDH	120–250 IU/mL	111	296 (236–359)	59	484 (372–666)	18	492 (457–844)	0.0001
Serum ferritin	5–150 µg/L	24	269 (163–925)	59	1430 (857–2395)	6	1420 (722–3100)	0.001
Serum CK	34–145 IU/mL	83	281 (104–1020)	26	84 (46–136)	17	100 (49–544)	0.0002
Serum CRP	<5 mg/L	107	190 (138–287)	55	152 (97–197)	28	179 (79–260)	0.02
Serum lactate	0.5–2.2 mmol/L	105	1.5 (1.1–2.3)	46	1.6 (1.3–2.5)	11	1.6 (1.1–5.0)	0.53
Elevated troponin ^b^	-	111	25/111 (23%)	6	3/6 (50%)	3	2/3 (67%)	0.06 ^b^

^a^ Includes individuals with confirmed Queensland tick typhus and scrub typhus. Median (IQR) or number (%) presented. ^b^ Troponin is expressed as a categorical variable as the Beckman Coulter assay for troponin I (normal reference range < 0.040 μg/L) was replaced by the Siemens Atellica assay (reference range < 20 ng/L normal for men; <10 ng/L normal for women) during the study period. INR: International Normalised Ratio; APTT: activated partial thromboplastin time; ALT: alanine aminotransferase; AST: aspartate aminotransferase; GGT: gamma glutamyl transferase; SAP: serum alkaline phosphatase, LDH: Lactate dehydrogenase; CK: creatinine kinase; CRP: c-reactive protein.

**Table 8:** Chest imaging on presentation and during admission to hospital.

Variable	Leptospirosis *n* = 109 ^b^	Q Fever *n* = 59 ^b^	Rickettsial Disease ^a^ *n* = 31 ^b^	*p*
Abnormal imaging on presentation	37 (34)	9 (15)	16 (52)	0.001
Any abnormal chest imaging during hospitalisation	64 (59)	18 (31)	20 (65)	0.001
Multilobar involvement	50 (46)	7 (12)	10 (32)	<0.0001
Alveolar changes	54 (49)	13 (22)	10 (32)	0.002
Interstitial changes	25 (23)	3 (5)	12 (39)	<0.0001
Pleural effusion	20 (18)	10 (17)	5 (16)	1.0

^a^ Includes individuals with confirmed Queensland tick typhus and scrub typhus. ^b^ 109 individuals with leptospirosis has chest imaging performed. The results of chest imaging were available in 58 individuals with Q fever and 31 individuals with rickettsial disease. Absolute number (%) presented.

**Table 9:** Clinical course of the patients in hospital.

Variable	Leptospirosis *n* = 111	Q Fever *n* = 59	Rickettsial Disease ^a^ *n* = 34	*p*
Length of hospital stay (days)	5 (3–8)	6 (3–11)	9 (4–15)	0.007
ICU admission	56 (50)	9 (15)	11 (32)	<0.0001
Length of ICU admission (days)	3 (2–5)	2 (1–8)	5 (3–18)	0.03
Vasopressor support at any point	56 (50)	6 (10)	9 (26)	<0.0001
Mechanical ventilation	14 (13)	1 (2)	6 (18)	0.01
RRT	18 (16)	0	3 (9)	0.007
Died	0	0	0	-

^a^ Includes individuals with confirmed Queensland tick typhus and scrub typhus. Median (interquartile range) or absolute number (%) presented, as appropriate. ICU: Intensive care unit; RRT: renal replacement therapy.

## Data Availability

Data cannot be shared publicly because of the Queensland Public Health Act 2005. Data are available from the Far North Queensland Human Research Ethics Committee (contact via email: fnq_hrec@health.qld.gov.au) for researchers who meet the criteria for access to confidential data.

## Supplementary Materials

The following supporting information can be downloaded at: https://www.mdpi.com/article/10.3390/tropicalmed11090237/s1, **Table S1:** Public Health Laboratory Network disease definitions of leptospirosis and acute Q fever [86,87]. **Table S2:** Definitions used for comorbidities in the cohort. **Table S3:** Demographic characteristics of the individuals with laboratory-confirmed leptospirosis, acute Q fever and rickettsial disease in the study. **Table S4:** Method of laboratory diagnosis. **Table S5:** Peak and nadir haematological and biochemical values recorded throughout hospital admission. **Figure S1:** Map of Far North Queensland, Australia, showing catchment area for current study. Adapted from Bird, K. et al. [88]. **Figure S2:** Proportion of individuals diagnosed with each disease by age group. **Figure S3:** Number of confirmed cases stratified by area of residence in FNQ. **File S1:** Methods for the polymerase chain reaction (PCR) for the identification of cases of Leptospirosis [89,90]. **File S2:** Methods for the polymerase chain reaction (PCR) for the identification of cases of *Coxiella burnetii* infection [91]. **File S3:** Methods for the polymerase chain reaction (PCR) for the identification of *Rickettsia* species [92,93] **File S4:** Methods for the polymerase chain reaction (PCR) for the identification of cases of *Orientia tsutsugamushi* infection [94].
