# Supplementary material for: Deciphering the “Doxycycline Deficiency Syndrome”: Defining the Characteristic Clinical, Laboratory and Imaging Findings in Patients Presenting with Leptospirosis, Q Fever and Rickettsial Diseases in Far North Queensland, Tropical Australia
Source: Trop Med Infect Dis. 2026 Aug 22;11(9):237. doi: 10.3390/tropicalmed11090237 (PMC13611417; doi:10.3390/tropicalmed11090237)
Supplement: Supplementary file 1 [file tropicalmed-11-00237-s001.zip › tropicalmed-4379378-supplementary.pdf]

Supplementary material

Table S1: Australian Public Health Laboratory Network disease definitions of leptospirosis and acute Q fever [1,2]

Leptospirosis	Definitive criteria<br>• Isolation of pathogenic *Leptospira* spp. from a clinical specimen; OR<br>• Fourfold or greater increase of *Leptospira* agglutination titre by Microscopic Agglutination Test (MAT) between acute and convalescent phase serum obtained at least two weeks apart OR<br>• A single *Leptospira* agglutination titre by MAT greater than or equal to 400 in one or more serum specimens; OR<br>• Detection of *Leptospira* DNA by nucleic acid test (NAT) from a clinical specimen.
Acute Q fever	Definitive Criteria<br>• Isolation of *C. burnetii*; OR<br>• Detection of *C. burnetii* by nucleic acid amplification test (NAAT); OR<br>• Seroconversion or 4-fold or greater increase in *C. burnetii* antibody titre to phase II antigen by complement fixation (CF) assay or by indirect immunofluorescent (IFA) IgG antibody assay in paired sera.

Table S2: Definitions of the comorbidities examined in the cohort

**Comorbidity [a]**	**Definition**
Diabetes mellitus	Documented in history, HbA1c > 6.5%, or on diabetic treatment
Chronic Cardiac Failure	Documented in history, on heart failure therapy, or an echocardiogram demonstrating LVEF< 50%)
Ischaemic heart disease	Documented history of a myocardial infarction or coronary artery bypass graft surgery
Chronic kidney disease	Documented in history or if baseline eGFR <90
Chronic lung disease	Documented in history; included interstitial lung disease, chronic obstructive pulmonary disease, bronchiectasis, cystic fibrosis, asthma or any other disease requiring therapy
Chronic liver disease	Documented as having cirrhosis
Active malignancy	Documented in history
Autoimmune condition	Documented in history: any condition documented with – or without – immunomodulatory therapy
Immunosuppression	If patient was taking any medications to suppress their immune system including regular corticosteroids, other immunosuppressants or immunomodulatory therapy
Hazardous alcohol intake	>10 standard drinks per week or >4 standard drinks in one day at least once/month
Smoker	Smoking tobacco on a regular basis

HbA1c: glycosylated haemoglobin; LVEF: left ventricular ejection fraction; eGFR: estimated glomerular filtration rate; CKD: chronic kidney disease.

[a] If documented in patient's medical history recorded by the admitting doctor

Table S3: Demographic characteristics of the individuals with laboratory-confirmed leptospirosis, acute Q fever and rickettsial disease in the study.

	Leptospirosis		Q fever		Rickettsial disease [a]		p
Variable	Number with data	All *n*=111	Number with data	All *n*=59	Number with data	All *n*=34	
Age (years) [b]	111	38 (24-55)	59	52 (42-62)	34	45 (36-64)	0.0006
Male gender [c]	111	94 (85)	59	44 (75)	34	21 (62)	0.01
Recorded comorbidity [c]	111	13 (12)	59	23 (39)	34	8 (24)	<0.0001
Interhospital transfer [c]	111	86 (77)	59	31 (53)	34	14 (41)	<0.0001

[a] Includes individuals with confirmed Queensland tick typhus and scrub typhus.

[b] Median (interquartile range)

[c] Absolute number (%)

Table S4: Method of laboratory diagnosis

Laboratory diagnosis	Leptospirosis	Q fever	Rickettsial diseases [a]
Acute serology	6 (5)	0	0
Convalescent serology	12 (11)	14 (24)	26 (76)
PCR	89 (90)	45 (76)	8 (24)
Culture	4 (4)	0	0
Total	111	59	34

Absolute number (%) presented. PCR: polymerase chain reaction.

[a] Includes individuals with confirmed Queensland tick typhus and scrub typhus.

Table S5: Peak and nadir haematological and biochemical values recorded throughout hospital admission

		Leptospirosis		Q fever		Rickettsial disease [a]		*p* [a]
Variable	Reference range	Number with data	All *n*=111	Number with data	All *n*=59	Number with data	All *n*=34	
Haemoglobin lowest	115 - 160 g/dL	111	112 (98-123)	59	116 (103-131)	34	102 (86-116)	0.0008
White cell highest	4.0 - 11.0 x10^9/L	111	12.8 (9.5-18.3)	59	8.8 (6.3-11.8)	34	11.8 (8.2-17.5)	0.0001
White cell lowest	4.0 - 11.0 x10^9/L	111	5.7 (4.2-7.8)	59	4.9 (3.8-5.7)	34	6.9 (4.7-9.3)	0.0002
Platelet count lowest	140 - 400 x10^9/L	111	84 (33-121)	58	91 (62-129)	34	76 (26-136)	0.41
Neutrophil highest	2.0 - 8.0 x10^9/L	111	10.7 (8.1-14.2)	58	5.0 (3.8-7.7)	34	9.0 (6.0-14.0)	0.0001
Neutrophil lowest	2.0 - 8.0 x10^9/L	111	3.5 (2.7-5.6)	58	2.8 (2.0-3.8)	34	4.4 (2.8-5.8)	0.0003
Lymphocyte lowest	1.0 - 4.0 x10^9/L	111	0.3 (0.2-0.5)	59	0.8 (0.5-1.1)	34	0.7 (0.4-1.4)	0.0001
INR highest	0.9 - 1.2	85	1.2 (1.1-1.3)	35	1.2 (1.1-1.3)	26	1.2 (1.1-1.5)	0.26
APTT highest	25 - 38 seconds	85	32 (30-36)	33	33 (30-42)	25	35 (33-45)	0.01
Lowest serum sodium	135- 145 mmol/L	111	132 (129-135)	59	130 (127-134)	33	131 (127-134)	0.28
Highest serum potassium	3.5 - 5.2 mmol/L	111	4.4 (4.0-4.9)	59	4.4 (4.1-4.8)	33	4.5 (4.2-4.9)	0.90
Highest serum urea	2.5 – 7.8 mmol/L	111	10.4 (6.1-27.0)	59	6.8 (4.7-9.2)	34	7.8 (4.6-17.2)	0.0003
Highest serum creatinine	45 - 90 µmol/L	111	179 (102-382)	59	88 (76-111)	34	120 (82-213)	0.0001
Lowest serum bicarbonate	22 - 32 mmol/L	111	20 (17-22)	59	23 (21-24)	33	21 (17-23)	0.0001
Highest serum bilirubin	<20 µmol/L	111	26 (19-48)	59	22 (16-48)	34	22 (14-43)	0.47
Highest serum ALT	<34 IU/ml	111	121 (68-208)	59	172 (122-332)	34	127 (109-207)	0.001
Highest serum AST	<31 IU/ml	111	131 (74-210)	59	196 (123-310)	34	179 (136-282)	0.0006
Highest serum GGT	<38 IU/ml	111	135 (70-235)	59	159 (61-320)	34	117 (66-318)	0.45
Highest serum SAP	30 - 110 IU/ml	111	146 (109-208)	59	157 (114-286)	34	173 (107-351)	0.12
Highest serum LDH	120 – 250 IU/ml	111	400 (327-534)	59	534 (408-701)	18	520 (472-906)	0.0001
Highest serum ferritin		24	310 (183-966)	21	1430 (857-2395)	6	1420 (722-3100)	0.001
Highest serum CK	34-145 IU/ml	83	350 (114-1020)	26	84 (46-136)	17	100 (49-629)	0.0001
Highest serum CRP	<5 mg/L	107	227 (159-323)	55	162 (110-227)	28	187 (90-260)	0.002
Highest serum lactate	0.5 - 2.2 mmol/L	105	2.0 (1.4-2.7)	46	2.2 (1.5-3.0)	11	3.2 (2.7-7.1)	0.005
Elevated serum troponin [b]	-	111	29/111 (26%)	6	3/6 (50%)	3	2/3 (67%)	0.06 [b]

Median (IQR) or absolute number (%) presented. [a] Includes individuals with confirmed Queensland tick typhus and scrub typhus. [b] Troponin is expressed as a categorical variable as the Beckman Coulter assay for troponin I (normal reference range <0.040 μg/L) was replaced by the Siemens Atellica assay (reference range <20 ng/L normal for men; <10 ng/L normal for women) during the study period.

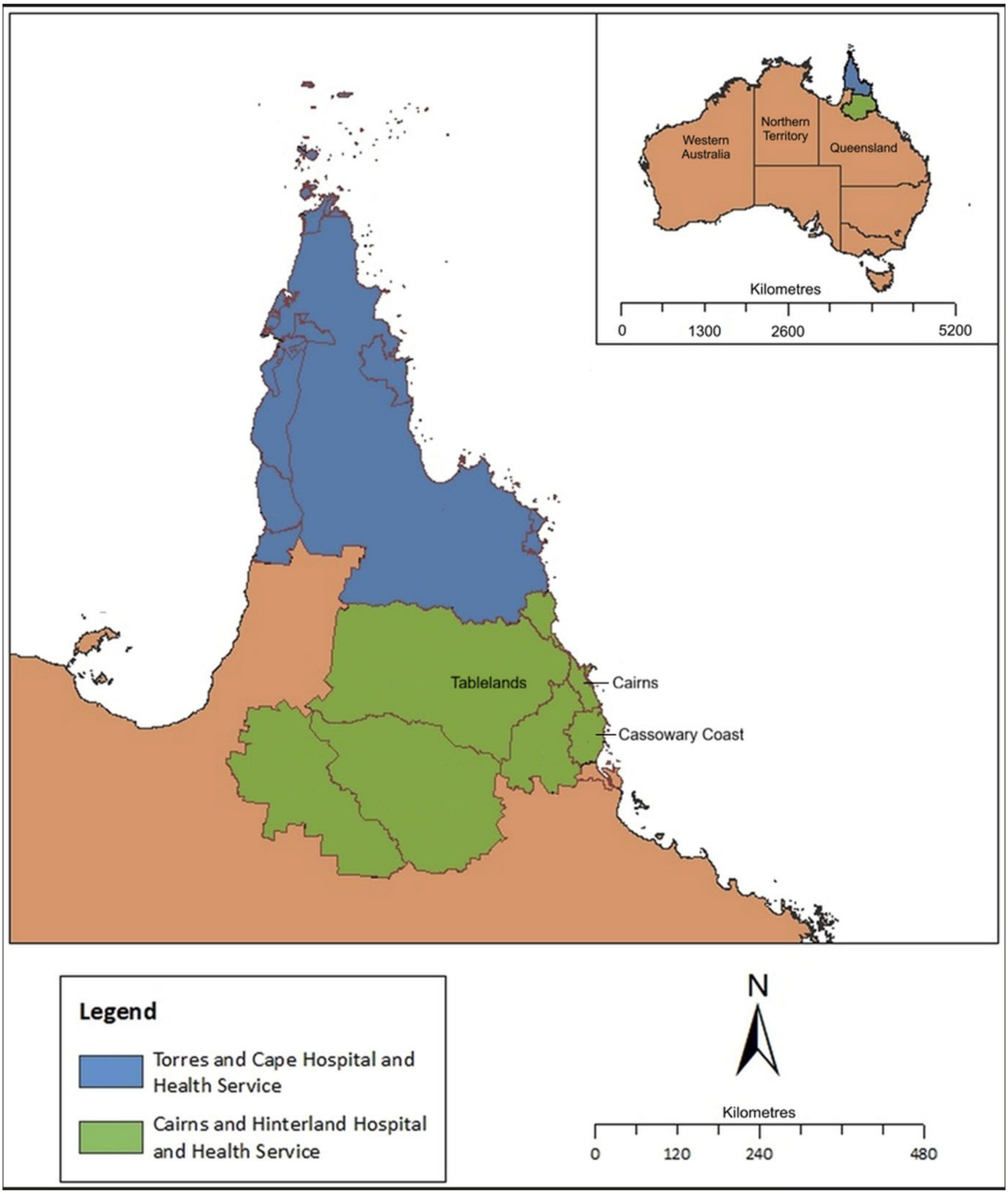

Figure S1: Map of Far North Queensland, Australia, showing catchment area for current study. Adapted from Bird, K. *et al* [3]

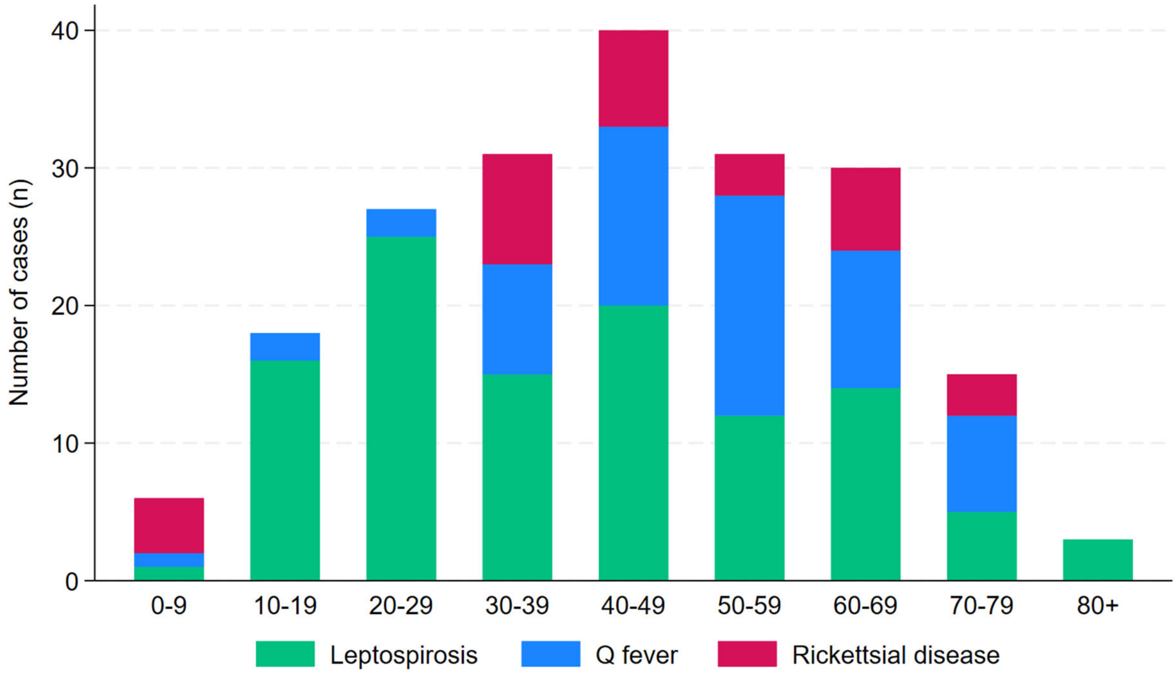

Figure S2: Proportion of individuals diagnosed with each disease by age group
Rickettsial disease includes individuals with confirmed Queensland tick typhus and scrub typhus.

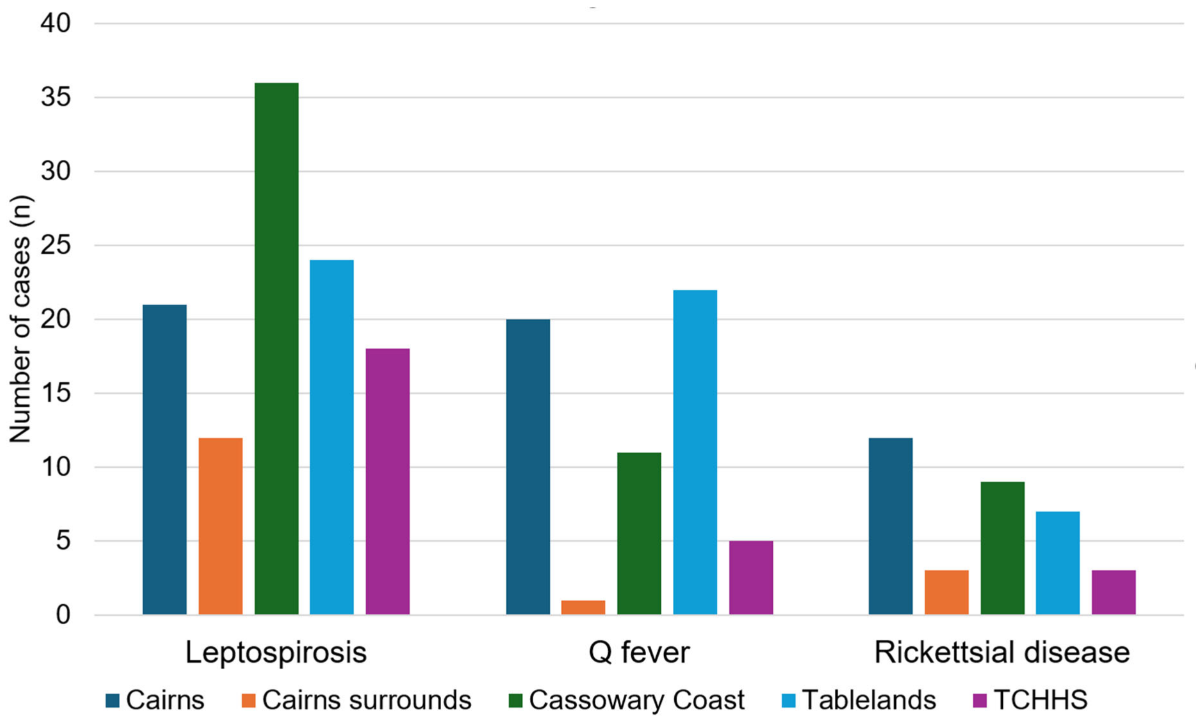

Figure S3: Number of confirmed cases stratified by area of residence in FNQ

Rickettsial disease includes individuals with confirmed Queensland tick typhus and scrub typhus.

TCHHS: Torres and Cape Hospital and Health Service which includes Cape York Peninsula and the Torres Strait Islands (see Figure S1).

File S1. Methods for the polymerase chain reaction (PCR) for the identification of cases of Leptospirosis.

Performed at Australia's national Leptospirosis Reference Laboratory in Brisbane, the World Health Organization (WHO) Collaborating Centre for Reference and Research on Leptospirosis for the Western Pacific Region:

https://www.health.qld.gov.au/public-health/forensic-and-scientific-services/testing-analysis/disease-investigation-and-analysis/leptospirosis-reference-laboratory

These assays are optimised in house, so these are the details for the exact validated methods used for the assays in the lab:

**PCR Assay target**	**16S (*rrs*)**	***lipL*32**
Years in use	2015 to April 2024	April 2024 to current
Extraction Platform	QIAGEN EZ1 / EZ2 Connect	QIAGEN EZ2 Connect
Extraction Kit	QIAGEN EZ1&2 Virus mini kit V2.0	QIAGEN EZ1&2 Virus mini kit V2.0
Specimen type	SST (sera)	SST (sera). Whole blood
PCR Reference Paper	Smythe *et al* 2002	Stoddard *et al* 2009
PCR Target	16S (*rrs* gene)	lipL32
Forward Primer sequence	5'-CCCGCGTCCGATTAG-3'	5'- AAGCATTACCGCTTGTGGTG -3'
Reverse Primer sequence	5'-TCCATTGTGGCCGRACAC-3'	5'- GAACTCCCATTTCAGCGATT -3'
Probe sequence	5'FAM-CTCACCAAGGCGACGATCGGTAGC-BHQ3'	5'- HEX-AAAGCCAGGACAAGCGCCG -BHQ1 -3'
PCR Instrument	QIAGEN Rotor-Gene Q	QIAGEN Rotor-Gene Q

Both assays accompanied by a second reaction; an equine herpes virus (EHV) extraction control assay to demonstrate successful DNA extraction of clinical specimens prior to PCR. Both assays are optimised for use and run simultaneously on the QIAGEN Rotor-Gene Q instrument

Papers that reference the assays (PCR primers and probes for these targets):

Stoddard RA, *et al* [4] and Smythe LD, *et al* [5]

Accreditation: https://nata.com.au/accredited-organisation/public-and-environmental-health-reference-laboratories-pehrl-micro-virology-41-13096/

File S2: Methods for polymerase chain reaction (PCR) for identification of cases of *Coxiella burnetii* infection

Performed at the Infectious Diseases Laboratory, Pathology Queensland, in Brisbane, Australia. Prior to January 2023 the assay was performed at the Public and Environmental Health Reference Laboratory, Pathology Queensland, in Brisbane, Australia. Both are National Association of Testing Authorities (NATA) accredited laboratories, conforming to the ISO 15189 standard.

These assays are optimised in house, so these are the details for the exact validated methods used for the assays in the lab:

PCR Assay target	IS1111a
Years in use	2017 to current [a]
Extraction Platform	QIAGEN QIAamp[#]
Extraction Kit	QIAGEN QIAamp Viral RNA Extraction kit [b]
Specimen type	Serum, EDTA blood, tissues
PCR Reference Paper	Banazis *et al*. 2010
PCR Target	*C. burnetii* transposase (IS1111a) gene
Forward Primer sequence	5'- GTTTCATCCGCGGTGTTAAT-3'
Reverse Primer sequence	5'- TGCAAGAATACGGACTCACG-3'
Probe sequence	5'-6FAM-CCCACCGCTTCGCTCGCTAA-BHQ1-3'
PCR Instrument	Bioer LineGene 9600

[a] Every patient in the series with a PCR diagnosis was diagnosed after 2017

[b] When extracting greater than 24 samples high-throughput automated nucleic acid extraction is performed using MGISP-960

Each sample is also tested using an RNase P (RNP) RT-PCR, which acts as an endogenous internal control (EIC) to ensure amplifiable intracellular nucleic acids are produced during extraction.

Reference paper for the assay (PCR primers and probes for the target):

Banazis MJ, *et al*.[6]

File S3: Methods for polymerase chain reaction (PCR) for identification of *Rickettsia* species

Performed at Infectious Diseases Laboratory, Pathology Queensland, in Brisbane, Australia. Prior to June 2023 the assay was performed at the Public and Environmental Health Reference Laboratory, Pathology Queensland, in Brisbane, Australia. Both are National Association of Testing Authorities (NATA) accredited laboratories, conforming to the ISO 15189 standard.

These assays are optimised in house, so these are the details for the exact validated methods used for the assays in the lab:

PCR Assay target	17 kDa antigen
Years in use	2011 to current [a]
Extraction Platform	QIAGEN QIAamp [b]
Extraction Kit	QIAGEN QIAamp Viral RNA Extraction kit [b]
Specimen type	EDTA whole blood, serum
PCR Reference Papers	Webb *et al* 1990, Jiang *et al* 2012
PCR Target	17kDa antigen gene
Forward Primer sequence [Rick2016TM-F]*	5'- GGGYGGTATGAAYAAACAAG-3'
Forward Primer sequence [Rfelis-2017For]	5'- AGGCGGTATGAATAAACAAG-3'
Reverse Primer sequence [Rick2016TM-R]*	5'- CCTACACCTACTCCVACAAG-3'
Probe sequence*	5'-6FAM- CCGAAYTGAGAACCAAGTAATGC-MGB-3'
PCR Instrument	Bioer LineGene 9600 / Applied Biosystem™ 7500

[a] Every patient diagnosed with rickettsial disease in the cohort was diagnosed after 2010

[b] When extracting greater than 24 samples high-throughput automated nucleic acid extraction is performed using MGISP-960

Degenerate bases are shown in red and underlined; Y-cytosine or thymine; V-adenine, cytosine or guanine

Each sample is also tested using an RNase P (RNP) RT-PCR, which acts as an endogenous internal control (EIC) to ensure amplifiable intracellular nucleic acids are produced during extraction.

Reference papers used to develop the assay (PCR primers and probes for these targets):

Jiang J, *et al*.[7] and Webb L, *et al*.[8]

File S4: Methods for polymerase chain reaction (PCR) for identification of cases of *Orientia tsutsugamushi*

Performed at Infectious Diseases Laboratory, Pathology Queensland, in Brisbane, Australia. Prior to June 2023 the assay was performed at the Public and Environmental Health Reference Laboratory, Pathology Queensland, in Brisbane, Australia. Both are National Association of Testing Authorities (NATA) accredited laboratories, conforming to the ISO 15189 standard.

These assays are optimised in house, so these are the details for the exact validated methods used for the assays in the lab:

PCR Assay target	47-kDa htr
Years in use	2011 to current [a]
Extraction Platform	QIAGEN QIAamp [b]
Extraction Kit	QIAGEN QIAamp Viral RNA Extraction kit [b]
Specimen type	EDTA whole blood, serum
PCR Reference Paper	Kawamori *et al.* 1993
PCR Target	47 kDa outer membrane protein antigen/high-temperature requirement A gene (47-kDa htr)
Forward Primer sequence	5'- AACTGATTTTATTCAAACTAATGCTGCT -3'
Reverse Primer sequence*	5'- TATGCCTGAGTAAGATACRTGAATRGAATT -3'
Probe sequence	5'-CY5-TGGTGGVCCGATGTTTAATCTTGAAGGA-BHQ2 -3'
PCR Instrument	Bioer LineGene 9600 / Applied Biosystem™ 7500

[b] When extracting greater than 24 samples high-throughput automated nucleic acid extraction is performed using MGISP-960

Degenerate bases are shown in red and underlined; R-adenine or guanine at this position.

Each sample is also tested using an RNase P (RNP) RT-PCR, which acts as an endogenous internal control (EIC) to ensure amplifiable intracellular nucleic acids are produced during extraction.

Paper that references the assays (PCR primers and probes for these targets)

Kawamori F, *et al.*[9]

References for supplementary files

1. *Public Health Laboratory Network. Leptospirosis - Laboratory case definition. https://www.cdc.gov.au/system/files/2025-09/leptospirosis-laboratory-case-definition_0.pdf.* Accessed on 30 April, 2026
2. Public Health Laboratory Network. Q fever (Coxiella burnetii) Laboratory case definition. https://www.cdc.gov.au/system/files/2025-09/q-fever-laboratory-case-definition_0_0.pdf. Accessed on 30 April, 2026
3. Bird, K.; Bohanna, I.; McDonald, M.; Wapau, H.; Blanco, L.; Cullen, J.; McLucas, J.; Forbes, S.; Vievers, A.; Wason, A.; et al. A good life for people living with disability: the story from Far North Queensland. *Disabil Rehabil* **2024**, *46*, 1787-1795, doi:10.1080/09638288.2023.2205172.
4. Stoddard, R.A.; Gee, J.E.; Wilkins, P.P.; McCaustland, K.; Hoffmaster, A.R. Detection of pathogenic Leptospira spp. through TaqMan polymerase chain reaction targeting the LipL32 gene. *Diagn Microbiol Infect Dis* **2009**, *64*, 247-255, doi:10.1016/j.diagmicrobio.2009.03.014.
5. Smythe, L.D.; Smith, I.L.; Smith, G.A.; Dohnt, M.F.; Symonds, M.L.; Barnett, L.J.; McKay, D.B. A quantitative PCR (TaqMan) assay for pathogenic Leptospira spp. *BMC Infect Dis* **2002**, *2*, 13, doi:10.1186/1471-2334-2-13.
6. Banazis, M.J.; Bestall, A.S.; Reid, S.A.; Fenwick, S.G. A survey of Western Australian sheep, cattle and kangaroos to determine the prevalence of Coxiella burnetii. *Vet Microbiol* **2010**, *143*, 337-345, doi:10.1016/j.vetmic.2009.12.002.
7. Jiang, J.; You, B.J.; Liu, E.; Apte, A.; Yarina, T.R.; Myers, T.E.; Lee, J.S.; Francesconi, S.C.; O'Guinn, M.L.; Tsertsvadze, N.; et al. Development of three quantitative real-time PCR assays for the detection of Rickettsia raoultii, Rickettsia slovaca, and Rickettsia aeschlimannii and their validation with ticks from the country of Georgia and the Republic of Azerbaijan. *Ticks Tick Borne Dis* **2012**, *3*, 327-331, doi:10.1016/j.ttbdis.2012.10.004.
8. Webb, L.; Carl, M.; Malloy, D.C.; Dasch, G.A.; Azad, A.F. Detection of murine typhus infection in fleas by using the polymerase chain reaction. *J Clin Microbiol* **1990**, *28*, 530-534, doi:10.1128/jcm.28.3.530-534.1990.
9. Kawamori, F.; Akiyama, M.; Sugieda, M.; Kanda, T.; Akahane, S.; Yamamoto, S.; Ohashi, N.; Tamura, A. Two-step polymerase chain reaction for diagnosis of scrub typhus and identification of antigenic variants of Rickettsia tsutsugamushi. *J Vet Med Sci* **1993**, *55*, 749-755, doi:10.1292/jvms.55.749.
